# In Silico and In Vitro Analysis of *IL36RN* Alterations Reveals Critical Residues for the Function of the Interleukin-36 Receptor Complex

**DOI:** 10.1016/j.jid.2023.06.191

**Published:** 2023-12

**Authors:** Niina K. Hassi, Timir Weston, Giulia Rinaldi, Joseph C. Ng, Asma Smahi, Sophie Twelves, Camilla Davan-Wetton, Dana Fakhreddine, Franca Fraternali, Francesca Capon

**Affiliations:** 1 Department of Medical and Molecular Genetics, School of Basic and Medical Biosciences, King's College London, London, United Kingdom; 2 Randall Centre for Cell & Molecular Biophysics, School of Basic and Medical Biosciences, King’s College London, London, United Kingdom; 3 Institute of Structural and Molecular Biology, University College London, London, United Kingdom; 4 IMAGINE Institute INSERM UMR 1163, Paris, France

## Abstract

Generalized pustular psoriasis is a potentially life-threatening skin disease, associated with *IL36RN* disease alleles. *IL36RN* encodes the IL-36 receptor antagonist (IL-36Ra), a protein that downregulates the activity of IL-36 cytokines by blocking their receptor (IL-36R). Although generalized pustular psoriasis can be treated with IL-36R inhibitors, the structural underpinnings of the IL-36Ra/IL-36R interaction remain poorly understood. In this study, we sought to address this question by systematically investigating the effects of *IL36RN* sequence changes. We experimentally characterized the effects of 30 *IL36RN* variants on protein stability. In parallel, we used a machinelearning tool (Rhapsody) to analyze the IL-36Ra three-dimensional structure and predict the impact of all possible amino acid substitutions. This integrated approach identified 21 amino acids that are essential for IL-36Ra stability. We next investigated the effects of *IL36RN* changes on IL-36Ra/IL-36R binding and IL-36R signaling. Combining invitro assays and machine learning with a second program (mCSM), we identified 13 amino acids that are critical for IL-36Ra/IL36R engagement. Finally, we experimentally validated three representative predictions, further confirming the reliability of Rhapsody and mCSM. These findings shed light on the structural determinants of IL-36Ra activity, with potential to facilitate the design of new IL-36 inhibitors and aid the interpretation of *IL36RN* variants in diagnostic settings.

## Introduction

IL-36α, IL36-β, and IL36-γ are three IL-1 family cytokines that signal through a common receptor (IL-36R). Upon binding with agonist, IL-36R associates with its accessory subunit (IL-1RacP), triggering a signaling cascade that culminates with the transcription of inflammatory genes, such as *IL6* and *IL8* (Bassoy et al., 2018).

The activity of IL-36 cytokines is modulated by the IL-36 receptor antagonist (IL-36Ra). This protein also binds IL-36R but prevents its association with IL-1RacP, thus inhibiting the activation of downstream pathways (Bassoy et al., 2018).

Because IL-36 cytokines are mostly active at barrier sites (skin, gut, and lung), effective IL-36Ra/IL-36R binding is critical for epithelial immune homeostasis (Han et al., 2020). In fact, loss-of-function variants of the gene encoding IL-36Ra (*IL36RN*) are associated with generalized pustular psoriasis (GPP), a potentially life-threatening skin disorder presenting with recurrent pustular eruptions and systemic upset (Marrakchi et al., 2011; Onoufriadis et al., 2011).

The discovery of *IL36RN* disease alleles has informed the clinical development of a new class of biologics that restore skin immune homeostasis by blocking IL-36R activation (Maçães et al., 2022). After two successful clinical trials (Bachelez et al., 2019, 2021), the anti-IL36R antibody spesolimab was granted Food and Drug Administration Breakthrough Therapy Designation, leading to its recent approval for the treatment of GPP (Blair, 2022). While spesolimab is also being investigated in hidradenitis suppurativa (Hwang et al., 2022) and atopic dermatitis (Bissonnette et al., 2023), other IL-36 inhibitors and approaches to IL-36 blockade are actively being researched (Todorović et al., 2019; Zhukov et al., 2022).

In this rapidly evolving landscape, a rigorous characterisation of *IL36RN* alterations could identify amino acid residues that are key to the function of the IL-36Ra/IL-36R complex, with the potential to inform further advances in drug design. The impact of *IL36RN* alleles, however, has not been fully investigated, with most functional studies focusing on two recurrent disease alleles that are only observed in North-African (p.Leu27Pro) and European (p.Ser113Leu) populations (Marrakchi et al., 2011; Onoufriadis et al., 2011; Tauber et al., 2016). Because the IL-36Ra structure has not been experimentally resolved, studies of variant effects have also been hindered by an incomplete understanding of the protein three-dimensional conformation.

In this study, we have addressed this issue by exploiting the seminal work of the DeepMind AlphaFold2 project, which has recently inferred >200M highly accurate protein structures (Jumper et al., 2021; Varadi et al., 2022). By combining the computational analysis of AlphaFold2 structures with the experimental characterization of mutant constructs, we have systematically assessed the effects of *IL36RN* sequence changes. This integrated approach has enabled us to identify key residues that are essential to IL-36Ra stability and IL-36Ra/IL-36R binding.

## Results

### Variant selection

**Figure 1:**
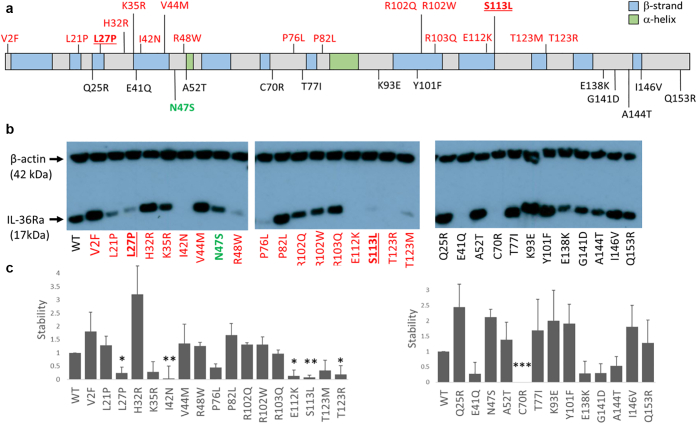
**Effects of *IL36RN* sequence variants on protein stability.** (**a**) Schematic showing the position of the examined variants. Changes observed in affected individuals are highlighted in red, with the recurrent p.Pro27Leu and p.Ser113Leu mutations in underlined font. The common p.Asn47Ser variant is shown in green, whereas rare population variants from the gnomAD database are in black. (**b**) Representative western blots showing the accumulation of wild-type (WT) and mutant IL-36Ra, after the transfection of the relevant cDNA constructs into HeLa cells. (**c**) Densitometry results for patient (left) and population (right) variants. Stability was calculated as the IL-36Ra/β-actin ratio normalised to wild-type values. Results are presented as means ± SD for 3 independent transfections. ∗*P*<0.05; ∗∗*P*<0.01; ∗∗∗*P*<0.001.

To explore the impact of missense alleles on IL-36Ra function, we examined 30 variants distributed along the entire protein sequence (Figure 1a). These variants included 17 rare changes observed in GPP cases (patient variants, Supplementary Table S1), 12 rare changes randomly selected from the gnomAD Database (population variants, Supplementary Table S2) and one common change detected in >10% of the East Asian sub-population (p.Asn47Ser).

### Variant effects on protein stability

We first investigated the effect of *IL36RN* variants on protein folding and stability. We generated mutagenized constructs for the 30 selected changes and overexpressed each cDNA in HeLa cells. We then measured protein accumulation by western blotting.

These experiments confirmed that p.Pro27Leu and p.Ser113Leu alleles, previously characterised as destabilizing changes (Marrakchi et al., 2011; Onoufriadis et al., 2011), were associated with a marked reduction in IL-36Ra levels (>4-fold decrease in protein accumulation compared with wild-type construct). A similar effect was observed for three additional GPP-associated alleles (p.Ile42Asn, p.Glu112Lys, and p.Thr123Arg) (Figure 1b and c).

**Figure 2:**
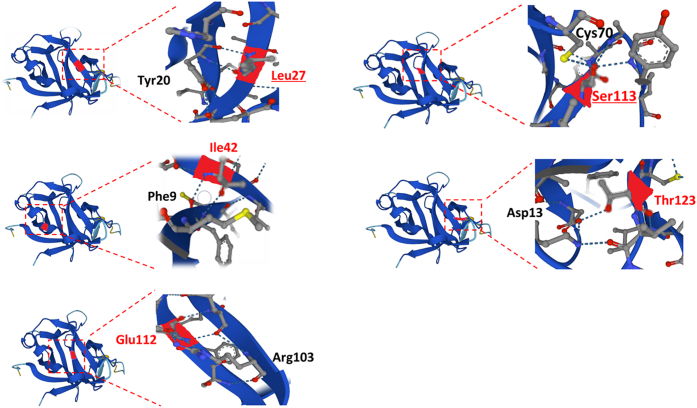
**Mapping destabilising changes to the three-dimensional IL-36Ra structure.** Each panel shows the position of the residues affected by mutations (left) with the enlarged view (right) displaying the hydrogen bonds formed with neighboring amino acids (blue dotted lines on structures). All residues except Thr123 map to β-strands (blue ribbons) within the protein core.

The five residues affected by the destabilizing changes were under strong evolutionary constraint, showing higher Genomic Evolutionary Rate Profiling scores (Davydov et al., 2010) than the population variants (average Genomic Evolutionary Rate Profiling score: 1.69 vs −0.3; *P* = 0.038). Although four of the five (80%) changes mapped to β-sheets within the protein core (Figure 2), only one of 13 population variants (7.7%) was found in a similar location (*P* = 0.008; Fisher exact test).

Only one population variant (p.Cys70Arg) had destabilizing effects. Interestingly, the amino acid affected by this change forms a hydrogen bond with Ser113, the target of the recurrent p.Ser113Leu mutation (Figure 2). Thus, the Ser113/Cys70 interaction is likely to be important for stable IL-36Ra folding.

**Figure 3:**
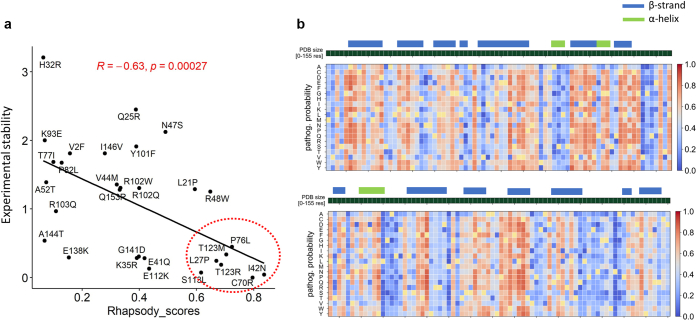
**Rhapsody pathogenicity predictions.** (**a**) Pathogenicity probabilities calculated by Rhapsody (Rhapsody scores) demonstrate a significant correlation with experimental measurements of protein stability (calculated as in 1c). Destabilizing changes generating high Rhapsody scores are highlighted with a red circle. (**b**) Heatmap illustrating the results of in silico saturation mutagenesis. Each column shows the Rhapsody pathogenicity probabilities for all possible substitutions at a given residue. The series of dark green squares above the diagram represents the IL36Ra amino acid sequence, with α-helixes and β-strands highlighted by coloured bars.

To further explore these findings, we assessed the effect of destabilizing changes using Rapid High-Accuracy Prediction of SAV Outcome based on DYnamics (Rhapsody). This is a machine learning tool that predicts variant pathogenicity by considering structural features and intrinsic protein dynamics (e.g. local fluctuations in residue position) (Ponzoni et al., 2020). In this study, we used Rhapsody to predict the variant impacts of the 30 selected changes using the IL-36Ra structure generated by AlphaFold2. We found that all destabilizing changes were associated with high (>50%) likelihoods of pathogenicity (Supplementary Table S1). We also observed an overall correlation between Rhapsody scores and experimental measurement of protein stability (r = −0.63; *P* = 0.0003) (Figure 3a).

**Supplementary Figure S1:**
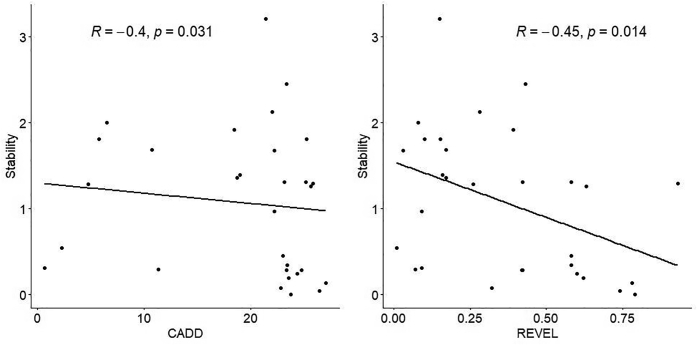
**Correlation between the experimental stability measurements reported in**Figure 1**c and the pathogenicity predictions obtained with CADD and REVEL.** Each dot represents a mutant construct. The R value reported on top of each plot is the Spearman correlation coefficient.

Interestingly, sequence-based pathogenicity predictors did not perform equally well. Combined annotation dependent depletion (Rentzsch et al., 2019) classified most changes (25/30) as pathogenic, including the common p.Asn47Ser variant. Rare Exome Variant Ensemble Learner (Ioannidis et al., 2016), which has been described as an accurate variant effect predictor for autoinflammatory alterations (Accetturo et al., 2020), offered better discrimination between benign and damaging alleles (Supplementary Figure S1). However, it misclassified the best characterized *IL36RN* disease allele (p.Ser113Leu) (Onoufriadis et al., 2011; Tauber et al., 2016) as “likely benign.”

### Saturation mutagenesis identifies variants that are essential to IL-36Ra stability

Having established that Rhapsody is the tool that best recapitulates the effects of *IL36RN* variants on protein stability, we decided to use this program for in silico saturation mutagenesis. We simulated all possible amino acid substitutions for each of the 155 residues that form the IL-36Ra protein. We then calculated pathogenicity scores for each change (Figure 3b). This approach confirmed the pattern observed in our stability assays, demonstrating that the 15 IL-36Ra residues that are most intolerant to substitutions (top decile, Supplementary Table S3) are more likely to map to β-strands than the remaining amino acids (80.0% vs. 47.8%, *P* = 0.027). These residues are also less accessible to solvents compare with the rest of the amino acids (average fraction of solvent accessible surface area (QSASA): 0.21 vs. 0.38, *P* = 0.023), indicating a preferential localization within the protein core. In fact, eight of the top decile residues cluster to three spatially adjacent β-sheets spanning amino acids 56–61, 96–104, and 119–123 (Supplementary Table S3). Thus, the combination of in vitro and in silico methods has allowed us to identify key residues that are critical to the folding and stability of IL-36Ra.

### Variant effects on receptor binding affinity

**Supplementary Figure S2:**
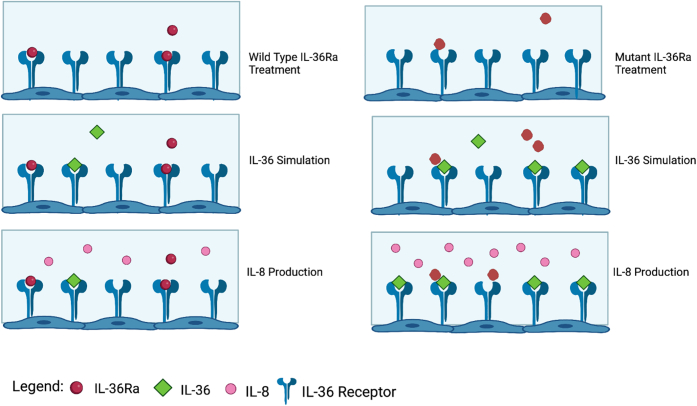
**Schematic representation of the IL-36Ra activity assay. HeLa-IL36R cells are treated with wild-type (left) or mutant (right) IL-36Ra protein.** The wild-type protein binds IL-36R and limits the amount of IL-8 that is produced when cells are stimulated with IL-36 (left). The mutant protein cannot engage with IL-36R, so that IL-36 stimulation results in enhanced IL-8 production (right). Created with biorender.com.

The majority of disease alleles that do not affect protein folding disrupt protein-protein interactions (Sahni et al., 2015). We, therefore, hypothesised that *IL36RN* variants mapping to the protein surface would destabilize the engagement of IL-36Ra with its receptor, favoring IL-36/IL-36R signaling over IL-36Ra/IL-36R binding. To validate this hypothesis, we treated HeLa cells with wild-type or mutant IL-36Ra, before stimulating the cultures with IL-36α. We then measured the production of IL-8 (Supplementary Figure S2). We selected this chemokine as a readout of IL-36/IL-36R signaling because it is potently induced by all IL-36 cytokines (Mahil et al., 2017; Swindell et al., 2018) and is markedly overexpressed in skin of patient with GPP (Baum et al., 2022). IL-8 is also downregulated after disease treatment with spesolimab or retinoids (Baumet al., 2022, Wang et al., 2018) (Supplementary Table S4). In fact, IL-8 plays a key role in driving neutrophilic inflammation (Matsushima et al., 2022), so that its induction has been the focus of various studies characterizing the effects of *IL36RN* disease alleles (Bal et al., 2019; Marrakchi et al., 2011; Tauber et al., 2016).

**Figure 4:**
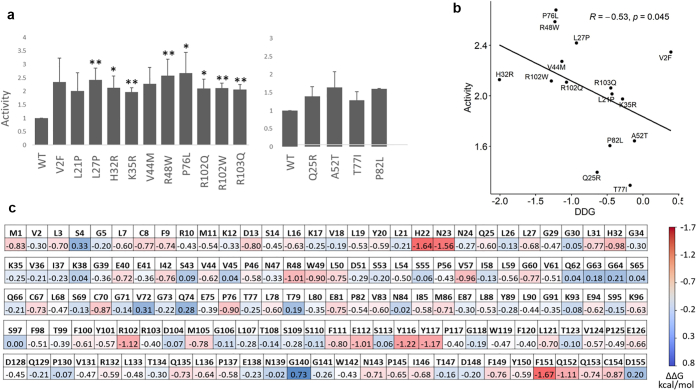
**Effects of *IL36RN* sequence variants on receptor binding affinity.** (**a**) Bar plot showing the effects of patient (left) and population (right) variants on IL-36 signaling (measured as IL-36 induced IL-8 production, normalised to wild-type). All results are presented as means ± standard deviation for 3 independent transfections. ∗*P*<0.05 and ∗∗*P*<0.01 for variants associated with at least 2-fold increase in IL-36 activity; (**b**) Changes in receptor binding affinity calculated by mCSM (ΔΔG values) demonstrate a significant correlation with experimental measurements of IL-36 signaling. (**c**) Heatmap illustrating the results of Ala scanning mutagenesis. Each cell shows the mCSM ΔΔG values for the relevant residue. ΔΔG, change in Gibbs free energy of binding (ΔG).

In this study, we measured IL-36 dependent IL-8 production to analyze the effects of 14 IL-36Ra surface changes (10 patient and 4 population variants) that did not destabilize the protein structure. We found that most alleles from the patients led to the upregulation of IL-36 signaling, whereas population variants did not (Figure 4a). In fact, the average IL8-fold induction was 2.25 for patient alleles versus 1.48 for population variants (*P* = 5.3 × 10^−5^).

The results of the activity assays did not correlate with the output of sequence-based pathogenicity predictors (combined annotation dependent depletion and Rare Exome Variant Ensemble Learner) or genomic evolutionary rate profiling scores (*P*>0.2 for all). Because Rhapsody has been developed for the analysis of single proteins, we sought an alternative, structure-based approach, which allowed us to model the effect of amino acid substitutions on the IL-36Ra/IL-36R interaction.

We first used protein-protein docking to model the structure of the IL-36Ra/IL-36R complex, based on that of its constituent proteins. We then analyzed the impact of *IL36RN* sequence variants using mCSM and mCSM-PPI2, two machine learning tools that predict the effects of missense changes on protein binding affinity (Pires et al., 2014; Rodrigues et al., 2019). This analysis showed that the impact of *IL36RN* alleles predicted by mCSM (the change in Gibbs free energy or ΔΔG) is significantly correlated with their effect on IL-36 activity (r = −0.53, *P* = 0.045) (Figure 4b).

Having established the reliability of its predictions, we used mCSM to systematically investigate which IL-36Ra residues can be altered without affecting receptor-ligand interactions. We undertook computational alanine scanning, a process in which the amino acids of a target protein are substituted with Ala to eliminate side-chain interactions while preserving the native structure of the protein. This showed that 91% of residue changes had a mild (−1<ΔΔG<0) or moderate (−2<ΔΔG<−1) destabilizing effect on the IL-36R/IL-36Ra interaction (Figure 4c). Interestingly, the 15 amino acids that are most intolerant to substitutions (top decile, Supplementary Table S3) include the residues that are affected by the p.His32Arg, p.Arg48Trp, p.Pro76Leu, p.Arg102Trp, and p.Glu112Lys disease alleles. These residues were more frequently found on the IL-36Ra/IL-36R binding interface than the remaining IL-36Ra amino acids (46.7% vs. 19.2%, *P* = 0.023).

### Validation of in silico predictions

Our correlation analyses suggest that protein stability is most accurately predicted with Rhapsody, whereas mCSM is the best tool to assess the impact of amino acid substitutions on IL-36Ra/IL-36R binding affinity.

To further confirm this, we selected representative predictions for experimental validation. We focused on the following three changes that are reported as variants of unknown significance in the ClinVar database of genomic variation: p.Cys67Phe (affecting a beta-helix in the protein core), p.Ala92Val (affecting a surface residue that does not map to the receptor binding interface), and p.Gln129Arg (affecting a surface residue that maps to the receptor binding interface).

**Figure 5:**
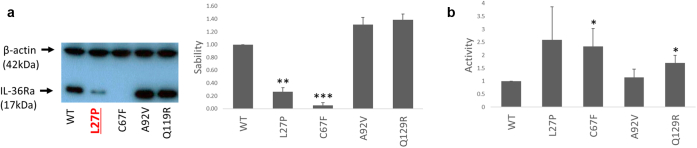
**Validation of bioinformatic predictions for selected changes.** (**a**) Left: representative western blot showing the accumulation of wild-type (WT) and mutant IL-36Ra, after the transfection of the relevant cDNA constructs into HeLa cells. The p.Pro27Leu disease allele was analyzed as a positive control and is highlighted in red underlined font. Right: Densitometry results. Stability was calculated as the IL-36Ra/β-actin ratio normalized to wild-type. (**b**) Bar plot showing the effects of selected variants on IL-36 signaling activity. All results are presented as means ± standard deviation for 3 independent transfections. ∗*P*<0.05; ∗∗*P*<0.01; ∗∗∗*P*<0.001.

Rhapsody predicts a deleterious effect for p.Cys67Phe (pathogenicity probability 0.77) but not for p.Ala92Val or p.Gln129Arg (pathogenicity probabilities 0.12 and 0.34, respectively). Accordingly, western blot analysis showed a reduction in protein accumulation for the p.Cys67Phe protein but not for the p.Ala92Val and p.Gln129Arg mutants (Figure 5a).

mCSM scores suggested that the effect of p.Ala92Val was modest (ΔΔG = −0.38 kcal/mol) and that of p.Gln129Arg was mildly destabilizing (ΔΔG = −0.65 kcal/mol). Our activity assay showed that p.Gln129Arg, but not Ala92Val, had an impact on IL-36 signaling (Figure 5b), verifying these predictions.

## Discussion

The aim of our study was to characterize the IL-36Ra residues that are essential for the stability of the protein and its interaction with IL-36R. We combined experimental and computational approaches, using the former to validate the performance of two well-established predictors of variant impact: Rhapsody, for prediction of damaging variant impacts in protein monomers, and mCSM, for prediction of changes in the stability of protein-protein interactions associated with variants. We then used these tools to systematically investigate the IL-36Ra structure generated by AlphaFold2.

A recent community assessment confirmed that pathogenicity predictions based on AlphaFold2 models were as accurate as those relying on experimental structures. AlphaFold2 models also performed better than homology-based ones, especially when the latter were derived from distantly related templates (Akdel et al., 2022).

Importantly, confidence metrics associated with individual amino acids were identified as an important parameter influencing the accuracy of variant effect prediction (Akdel et al., 2022). Thus, we are confident in our decision to use AlphaFold2 models for IL-36Ra (<5% of residues predicted with low confidence) but not for IL-36R (>15% of residues predicted with low or very low confidence).

Recent studies also showed that the AlphaFold2 system cannot directly infer the impact of amino acid changes on protein structure (Buel and Walters, 2022, Pak et al., 2023), supporting our strategy of using tools specifically designed for pathogenicity predictions. We specifically selected Rhapsody and mCSM, based on significant correlations with our experimental results and high performance with benchmark datasets (Pires et al., 2014; Ponzoni et al., 2020).

In keeping with experimental results obtained in other systematic studies of variant effects (Høie et al., 2022), our Rhapsody analysis showed that the amino acids that are essential to protein stability are mostly buried in the core. In this case, the observation is particularly noteworthy because IL-36Ra is a low molecular weight protein (17 kDa) with a small hydrophobic core. In this context, the p.Cys67Phe substitution examined in our validation experiment had a destabilizing effect. Although this change was identified in a single patient with GPP (Zea-Vera et al., 2019) and is currently classified as a variant of unknown significance, our computational and experimental findings indicate that it could be considered as a pathogenic mutation. In contrast, the p.Ala92Val and p.Gln129Arg surface substitutions are likely to be benign because mCSM scores and IL-36 activity assays point to very modest effects on receptor binding affinity. Thus, our observations support the application of Rhapsody and mCSM for the interpretation of *IL36RN* variants. Given that these tools outperformed sequence-based predictors in our analyses, their application could be particularly informative in diagnostic settings, in which *IL36RN* is often sequenced as a part of an autoinflammatory disease gene panel (Omoyinmi et al., 2017).

In our study, we also used Rhapsody for in silico saturation mutagenesis and mCSM for scanning of alanine. The former analysis uncovered clusters of hydrophobic residues (e.g., Phe98/Phe100; Pro117/Trp119/Leu121) that are essential to IL-36Ra folding and stability. These amino acids are likely to play an important role in maintaining the structure of IL-1 family cytokines, because they are broadly conserved in paralogous proteins, such as IL-1Ra, IL-36α, IL-36γ, and IL-38 (Wang et al., 2010). The cytokine IL-38, is of particular interest, given it can bind IL-36R and inhibit downstream signaling, similar to IL-36Ra (de Graaf et al., 2022).

Alanine scanning identified key hydrophilic amino acids (e.g., His22, Asn23, and Arg102) at the IL-36Ra/IL-36R binding interface. Notably, none of these residues are conserved in IL-38, even if their hydrophilic properties are preserved (the corresponding positions are: Arg23, Asp24, and Gln103) (de Graaf et al., 2022). In this context, our integrated in vitro/in silico approach could be applied to the study of IL-36R/IL-38 binding, with the potential to reveal the structural determinants of its inhibitory function. Given that the IL-38 signaling complex is yet to be crystalized, such studies could prove particularly informative.

While the effects of IL-36Ra have been more extensively investigated than those of IL-38 (Bassoy et al., 2018), the only experimentally resolved structure for IL-36R is that of the extracellular domain of the receptor, in complex with a spesolimab fragment (Larson et al., 2020). Thus, our dissection of IL-36Ra/IL-36R binding sheds light on the structural determinants of this interaction, with the potential to facilitate the design of novel IL-36 inhibitors for the treatment of skin inflammation.

## Materials and Methods

### IL-36Ra protein structure

The IL-36Ra protein structure generated by AlphaFold2 (Varadi et al., 2022) (AF-Q9UBH0-F1-model_v3.pdb) was obtained from the AlphaFold Protein Structure Database at https://alphafold.ebi.ac.uk. Predicted local-distance difference test scores were also retrieved, confirming that only 5 of 155 residue positions (3%) had been inferred with low confidence (50< predicted local-distance difference test <70). The structure was validated with MolProbity (Williams et al., 2018) (http://molprobity.biochem.duke.edu/).

### Modeling of the IL-36Ra/IL-36R complex

The AlphaFold2 IL-36R structure (AF-Q9HB29-F1-model_v3.pdb) was deemed unsuitable for analysis because >15% of residue positions had been inferred with low or very low (Predicted local-distance difference test <50) confidence. The three-dimensional structure of IL-36R was, therefore, derived by homology modeling. The IL-36R amino acid sequence (UniProt ID: Q9HB29) was used to search the Protein Data Bank (PDB, www.rcsb.org), using the Basic Local Alignment Search Tool (Altschul et al., 1990) (BLAST, https://blast.ncbi.nlm.nih.gov/Blast.cgi). The top hit (IL-1/IL-1Ra complex; PDB entry 1IRA) was aligned with IL-36Ra using T-Coffee (Notredame et al., 2000). The three-dimensional protein structure was then predicted with SWISS-MODEL (Waterhouse et al., 2018) and validated with MolProbity.

Next, three-dimensional models of IL-36Ra and IL-36R were aligned to the experimentally resolved structure of the IL-1/IL-1Ra complex (PDB ID: 1IRA), using PyMol (Schrodinger, NY). RosettaDock (Chaudhury et al., 2011) (https://r2.graylab.jhu.edu) was then used to infer the most energetically favorable model for the IL-36Ra/IL-36R complex. The predicted IL36-Ra/IL36R structure with the lowest interface energy score, overall root-mean-squared deviation and interface root-mean-squared deviation was selected for analysis with the tools listed below.

The programs mCSM (Pires et al., 2014) (http://biosig.unimelb.edu.au/mcsm/) and mCSM-PPI2 (Rodrigues et al., 2019) (https://biosig.lab.uq.edu.au/mcsm_ppi2/) were used to quantify the changes to a calculated score that is a proxy to the ΔΔG associated with each variant. The fraction of solvent accessible surface area (QSASA, equal to the quotient between solvent accessible surface and total surface) was calculated for each residue, using POPScomp (Fraternali and Cavallo, 2002) (http://popscomp.org:3838/). Amino acids mapping to the IL-36R/IL36Ra binding interface were identified with MutaBind2 (Zhang et al., 2020) (https://lilab.jysw.suda.edu.cn/research/mutabind2/).

### IL-36Ra stability assay

For protein stability assays, HeLa-IL36R cells were transfected with wild-type or mutant myc-IL36RN, using Lipofectamine 2000 (Life Technologies, Carlsbad, CA; catalogue n: 11668027). Transfected HeLa-IL36R cells were harvested after 18 hours, and protein extracts were analyzed using western blotting, as described in the Supplementary Methods.

### IL-36Ra activity assay

IL-36Ra proteins used in activity assays were generated by transfecting HEK293 cells with wild-type or mutant myc-IL36RN, using Lipofectamine 2000. After 24 hours, cells were harvested and recombinant proteins were isolated from lysates, using the c-Myc tagged protein mild purification kit (MBL International Corporation, Woburn, MA; catalogue n:3305).

HeLa-IL36R cells were first starved in supplement-free RPMI media for 4 hours and then treated with 300 ng purified IL-36Ra protein (wild-type or mutant, generated as described above). After 30 minutes, cultures were stimulated with 10 ng/ml IL36α (RD Systems, MN; catalogue n: 6995-IL). Culture supernatants were collected after 4 hours and analyzed by ELISA.

### Statistics

Correlation analyses were conducted with Spearman rank test. The characteristics of IL-36Ra proteins harboring different changes (wild-type vs. mutant sequence and population vs. patient variant) were compared using an unpaired *t*-test or Fisher exact test, as appropriate. All tests were conducted in R v4.1.1.

### Data availability statement

The authors confirm that the data supporting the findings of this study are available in the article and its Supplementary Materials.

## ORCIDs

Niina K Hassi: http://orcid.org/0000-0002-1548-1199

Timir Weston: http://orcid.org/0000-0002-9193-5578

Giulia Rinaldi: http://orcid.org/0000-0002-4212-4584

Joseph C Ng: http://orcid.org/0000-0002-3617-5211

Asma Smahi: http://orcid.org/0000-0002-6002-0981

Sophie Twelves: http://orcid.org/0000-0002-6568-2925

Camilla Davan-Wetton: http://orcid.org/0000-0003-1252-7136

Dana Fakhreddine: http://orcid.org/0000-0003-3521-7093

Franca Fraternali: http://orcid.org/0000-0002-3143-6574

Francesca Capon: http://orcid.org/0000-0003-2432-5793

## Conflict of Interests

FC has received research grants and consultancy fees from Boehringer Ingelheim.

## Figures and Tables

**Supplementary Figure S3:**
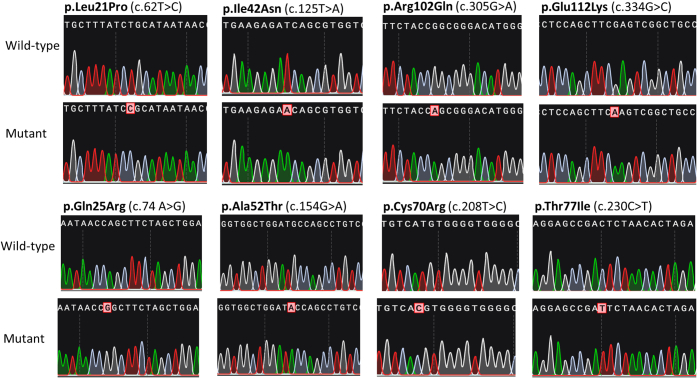
**Chromatograms of mutagenized constructs for representative *IL36RN* changes, including patient (top panel) and population (bottom panel) variants.**

**Supplementary Table S1:** Patient Variants Included in Functional Studies

Variant	Frequency^1^	Reference	Pathogenicity predictions		
			REVEL	CADD	Rhapsody
p.Val2Phe	0.00005	(Bal et al., 2019)	0.15	25.2	0.16
p.Leu21Pro	0.00003	(Ellingford et al., 2016)	0.93	25.8	0.59
p.Leu27Pro	0.001	(Marrakchi et al., 2011)	0.60	24.3	0.67
p.His32Arg	-	(Körber et al., 2013)	0.15	21.4	0.06
p.Lys35Arg	0.001	(Setta-Kaffetzi et al., 2013)	0.42	23.3	0.39
p.Ile42Asn	0.000009	(Takeichi etal., 2017)	0.74	26.4	0.84
p.Val44Met	0.00006	(Wang et al., 2016)	0.17	18.7	0.32
p.Arg48Trp	0.001	(Onoufriadis et al., 2011)	0.63	25.6	0.65
p.Pro76Leu	0.005	(Körber et al., 2013)	0.58	23.0	0.72
p.Pro82Leu	0.004	(Li et al., 2014)	0.03	22.2	0.13
p.Arg102Trp	0.005	(Setta-Kaffetzi et al., 2013)	0.58	23.1	0.33
p.Arg102Gln	0.000009	(Li et al., 2013)	0.42	25.1	0.40
p.Arg103Gln	0.00009	(Mössner et al., 2018)	0.09	22.2	0.11
p.Glu112Lys	0.00006	(Hayashi et al., 2014)	0.78	27.0	0.43
p.Ser113Leu	0.007	(Onoufriadis et al., 2011)	0.32	22.8	0.62
p.Thr123Arg	-	(Farooq et al., 2013)	0.62	23.5	0.69
p.Thr123Met	0.0001	(Kanazawa et al., 2013)	0.58	23.4	0.70

The recurrent p.Leu27Pro and p.Ser113Leu mutations are highlighted in bold font.

Abbreviation: CADD, Combined Annotation Dependent Depletion; REVEL, Rare Exome Variant Ensemble Learner.

1 Maximum frequency observed across gnomAD (r2.1.1) populations.

**Supplementary Table S2:** Population Variants Included in Functional Studies

Variant	rs identifier	Pathogenicity Predictions			Frequency^1^
		REVEL	CADD	Rhapsody	
p.Gln25Arg	rs867378394	0.43	23.3	0.39	-
p. Glu41Gln	rs771984756	0.42	24.7	0.42	0.00005
p.Asn47Ser	rs28938777	0.28	22.0	0.49	0.08
p.Ala52Thr	rs755465505	0.16	19.0	0.07	0.00005
p.Cys70Arg	rs375718709	0.79	23.7	0.80	0.00006
p.Thr77Ile	rs372880215	0.17	10.7	0.10	0.0001
p.Lys93Glu	rs746109701	0.08	6.5	0.07	0.00005
p.Tyr101Phe	rs769214649	0.39	18.4	0.39	0.0002
p.Glu138Lys	rs750580815	0.07	11.3	0.15	0.00003
p.Gly141Asp	rs758533837	0.09	0.7	0.40	0.00005
p.Ala144Thr	rs780261792	0.01	2.3	0.07	0.00003
p.Ile146Val	rs202059991	0.10	5.8	0.28	0.00009
p.Gln153Arg	rs771496493	0.26	4.8	0.33	0.00004

The common p.Asn47Ser variant is highlighted in bold font.

Abbreviation: CADD, Combined Annotation Dependent Depletion; REVEL, Rare Exome Variant Ensemble Learner.

1 Maximum frequency observed across gnomAD (r2.1.1) populations.

**Supplementary Table S3:** IL-36Ra Residues that are Most Intolerant to Substitutions

Rhapsody Stability Analysis		mCSM Affinity Analysis	
Residue	Pathogenicity Score^1^	Residue	Pathogenicity Score^2^
Phe98	0.81	Phe151	−1.67 (C)
Phe100	0.78	His22	−1.64 (C)
Cys122	0.78	Asn23	−1.56 (C)
Val44	0.76	Tyr116	−1.22
Trp119	0.75	Cys122	−1.17
Leu59	0.75	Arg102	−1.12 (C)
Leu121	0.74	Gln152	−1.10 (C)
Cys67	0.74	Arg48	−1.01
Pro56	0.73	Glu112	−1.01
Leu19	0.72	His32	−0.98 (C)
Gly34	0.72	Val57	−0.96
Ala6	0.72	Pro76	−0.90
Pro117	0.71	Trp49	−0.90
Gly5	0.71	Cys154	−0.87 (C)
Gly60	0.71	Cys70	−0.87

1 Average pathogenicity likelihood across all 19 possible substitutions.

2 ΔΔG measured by Ala scanning (kcal/mol); C, IL-36R/IL-36Ra contact residue.

**Supplementary Table S4:** Features of the Cytokines and Chemokines that are Upregulated by IL-36

Gene	Fold Up-Regulation in Primary Keratinocytes Treated with^1^			Upregulated in GPP skin^2^	Downregulated by GPP treatment	
	IL-36α	IL-36β	IL-36γ	-	Skin^3^	Blood^4^
*IL36G*	17.7	23.8	21.4	Yes	Yes	No
*CXCL1*	7.8	9.2	9.9	Yes	Yes	Yes
*CCL20*	7.6	9.0	9.2	Yes	No	No
*CXCL8*	7.0	8.5	8.8	Yes	Yes	Yes
*IL32*	5.6	5.7	5.6	No	No	Yes
*IL1B*	5.1	5.0	5.3	Yes	Yes	Yes
*CXCL5*	2.6	3.8	3.4	No	Yes	No
*IL36RN*	2.5	3.8	3.2	Yes	No	No
*CXCL3*	2.5	3.3	2.9	No	Yes	Yes
*CXCL2*	2.6	3.0	2.9	Yes	Yes	No
*IL1A*	2.8	2.3	2.5	No	No	No
*IL24*	2.6	2.2	2.6	No	Yes	No
*IL23A*	2.2	2.6	2.5	Yes	Yes	No
*CXCL16*	2.3	2.6	2.4	No	No	No
*IL20*	2.3	2.0	2.3	Yes	Yes	No

Abbreviation: GPP, generalized pustular psoriasis.

1 Cultured keratinocytes (Mahil et al., 2017).

2 GPP vs healthy skin (Baum et al., 2022).

3 GPP skin sampled before and after treatment with spesolimab (Baum et al., 2022).

4 GPP peripheral blood mononuclear cells sampled before and after treatment with acitretin (Wang et al., 2018).

**Supplementary Table S5:** Sequence of Mutagenesis Primers^1^

Variant	Primers
p.Val2Phe	CGCGATCGCCATGTTCCTGAGTGGGGC
	GCCCCACTCAGGAACATGGCGATCGCG
p.Leu21Pro	AGAAGCTGGTTATTATGCGGATAAAGCACCTTCAATGCC
	GGCATTGAAGGTGCTTTATCCGCATAATAACCAGCTTCT
p.Gln25Arg	GCCCTCCAGCTAGAAGCCGGTTATTATGCAGATAA
	TTATCTGCATAATAACCGGCTTCTAGCTGGAGGGC
p.Glu41Gln	GACCACGCTGATCTGTTCACCTTTAATGACCTTCCC
	GGGAAGGTCATTAAAGGTGAACAGATCAGCGTGGTC
p.Ile42Asn	GATTGGGGACCACGCTGTTCTCTTCACCTTTAATG
	CATTAAAGGTGAAGAGAACAGCGTGGTCCCCAATC
p.Val44Met	ACCGATTGGGGACCATGCTGATCTCTTCACC
	GGTGAAGAGATCAGCATGGTCCCCAATCGGT
p.Asn47Ser	CAGCCACCGACTGGGGACCACGCTGATC
	GATCAGCGTGGTCCCCAGTCGGTGGCTG
p.Arg48Trp	AGCGTGGTCCCCAATTGGTGGCTGGATGCCA
	TGGCATCCAGCCACCAATTGGGGACCACGCT
p.Ala52Thr	GGGACAGGCTGGTATCCAGCCACCGATTG
	CAATCGGTGGCTGGATACCAGCCTGTCCC
p.Cys67Phe	GGTGGAAGCCAGTTCCTGTCATGTGGG
	CCCACATGACAGGAACTGGCTTCCACC
p.Cys70Arg	GCCCCACCCCACGTGACAGGCACTG
	CAGTGCCTGTCACGTGGGGTGGGGC
p.Thr77Ile	GGTGGGGCAGGAGCCGATTCTAACACTAGAG
	CTCTAGTGTTAGAATCGGCTCCTGCCCCACC
p.Pro82Leu	GCTCCATGATGTTCACTAGCTCTAGTGTTAGAGTC
	GACTCTAACACTAGAGCTAGTGAACATCATGGAGC
p.Lys93Glu	GAAGCTCTTGGATTCCTCGGCACCAAGATAGAGCT
	AGCTCTATCTTGGTGCCGAGGAATCCAAGAGCTTC
p.Tyr101Phe	GTCCCGCCGGAAGAAGGTGAAGCTCTTGGA
	TCCAAGAGCTTCACCTTCTTCCGGCGGGAC
p.Arg102Gln	GCCCCATGTCCCGCTGGTAGAAGGTGAAG
	CTTCACCTTCTACCAGCGGGACATGGGGC
p.Arg103Gln	GAGCCCCATGTCCTGCCGGTAGAAGGT
	ACCTTCTACCGGCAGGACATGGGGCTC
p.Glu112Lys	GTAGGCAGCCGACTTGAAGCTGGAGGTGA
	TCACCTCCAGCTTCAAGTCGGCTGCCTAC
p.Ser113Leu	CCTCCAGCTTCGAGTTGGCTGCCTACCCGGG
	CCCGGGTAGGCAGCCAACTCGAAGCTGGAGG
p.Thr123Arg	GCTTCAGGCACCCTGCACAGGAACCAG
	CTGGTTCCTGTGCAGGGTGCCTGAAGC
p.Gln129Arg	CCTGAAGCCGATCGGCCTGTCAGACTCACCC
	GGGTGAGTCTGACAGGCCGATCGGCTTCAGG
p.Glu138Lys	CCAGCCACCATTCTTGGGAAGCTGGGTGA
	TCACCCAGCTTCCCAAGAATGGTGGCTGG
p.Gly141Asp	GGGGGCATTCCAGTCACCATTCTCGGG
	CCCGAGAATGGTGACTGGAATGCCCCC
p.Ala144Thr	CTGTGATGGGGGTATTCCAGCCACCATTCTCG
	CGAGAATGGTGGCTGGAATACCCCCATCACAG
p.Ile146Val	GGCTGGAATGCCCCCGTCACAGACTTCTACT
	AGTAGAAGTCTGTGACGGGGGCATTCCAGCC
p.Gln153Arg	GACTTCTACTTCCAGCGGTGTGACTAGGGCAAC
	GTTGCCCTAGTCACACCGCTGGAAGTAGAAGTC

1 The p.Pro27Leu, p.His32Arg, p.Lys35Arg, p.Pro76Leu, and p.Thr123Met constructs have been described elsewhere (Tauber et al., 2016).

## Supplementary Materials and Methods

### Variant selection, pathogenicity predictions, and evolutionary conservation analysis

dbSNP data (build 153) was accessed at https://www.ncbi.nlm.nih.gov/snp/. Precomputed CADD scores were retrieved from the database hosted at cadd.gs.washington.edu, whereas GERP and REVEL scores were obtained through the Ensembl genome browser (www.ensembl.org). Rhapsody was accessed at http://rhapsody.csb.pitt.edu. The IL-36Ra PDB file (AF-Q9UBH0-F1-model_v3) retrieved from the AlphaFold2 database was used as an input for single-variant analysis and saturation mutagenesis.

### Generation of mutagenized constructs

Constructs were generated using the QuikChange Lightning Site-Directed Mutagenesis Kit (Agilent Technologies, Santa Clara, CA; catalogue n. 210518) and primers designed with the QuikChange Primer Design tool (www.agilent.com/store/primerDesignProgram.jsp) (Supplementary Table S5). In brief, 10 ng wild-type construct was combined with 125 ng of each primer, 5 μl 10×QuickChange Lighting Buffer, 1 μl dNTP mix and 1.5 μl QuickSolution reagent in a 50 μl final volume. The reactions were incubated under the following cycling conditions: 2 minutes 95 °C, 18× (20 sec 95 °C, 10 sec 60 °C, and 2 min 68 °C), 5 minutes 68 °C. Next, the parental plasmid was digested with 2 μl DpnI for 5 minutes at 37 °C. Finally, the mutagenized plasmid was transformed into XL10-Gold ultracompetent cells and single-colonies were expanded. All constructs were validated by sequencing the entire *IL36RN* coding region, pCMV promoter, bovine growth hormone polyadenylation site and the myc sequence (Supplementary Figure S3).

### Cell culture

HEK293 cells were cultured in DMEM supplemented with GlutaMax-I, 50 U/ml of penicillin/50 μg per ml of streptomycin and 10% fetal calf serum (all from Life Technologies, Carlsbad, CA; catalogue n: 61965026, 15140122, and 10500064). HeLa-IL36R cells (kindly provided by Prof Seamus Martin, Trinity College Dublin, Ireland) were cultured in RPMI medium 1640 (Life Technologies; catalogue n: 21875034) supplemented as above.

### Western blotting and ELISA

Cell lysates were loaded on a 15% polyacrylamide gel and electrophoresed for 2 hours at 100 V. Separated proteins were transferred to a polyvinylidene fluoride membrane (Roche, Basel, Switzerland; catalogue n: 03010040001) and probed overnight at 4 °C with mouse anti c-myc (Santa Cruz Biotechnology, Dallas, TX; catalogue n: sc-40) and rabbit anti-β−actin (Cell Signalling Technology, Danvers, MA; catalogue n: 4967S) antibodies at a 1:1,000 dilution. The membrane was then incubated for 1 hour at room temperature with a horseradish peroxidase–conjugated secondary antibody (Polyclonal Anti-Mouse IgG (Agilent; catalogue n: P0447), or Polyclonal Anti-Rabbit IgG (GE Healthcare, Chicago, IL; catalogue n: NA934V)) diluted 1:10,000. Finally, the membrane was treated with the Amersham ECL Western Blotting Prime Detection Reagent (GE Healthcare; catalogue n: RPN2232) for 5 minutes. CL-Exposure autoradiography films (Thermo Fisher Scientific, Waltham, MA; catalogue n: 34090) were exposed to the membrane, developed using an automated film developer and analyzed with ImageJ (Schneider et al., 2012) to measure IL-36Ra to β-actin ratios.

IL-8 levels were measured with the DuoSet CXCL8/IL-8 ELISA kit (R&D Systems, Minneapolis, MN; catalogue n: DY208-05), using 1:40 dilutions of the culture supernatants.
